# Symptoms in individuals with adult-onset ADHD are masked during childhood

**DOI:** 10.1007/s00406-018-0893-3

**Published:** 2018-04-06

**Authors:** Hirotaka Kosaka, Toru Fujioka, Minyoung Jung

**Affiliations:** 1 Research Center for Child Mental Development, University of Fukui, 23-3 Matsuokashimoaizuki, Eiheiji, Fukui, 910-1193 Japan; 2 Department of Neuropsychiatry, University of Fukui, 23-3 Matsuokashimoaizuki, Eiheiji, Fukui, 910-1193 Japan

The continuity of neurodevelopmental disorders from childhood to adulthood has garnered much attention. However, three studies have recently examined the possibility that adult-onset attention-deficit hyperactivity disorder (ADHD) is a separate neurodevelopmental disorder from childhood-onset ADHD [1–3]. A cohort study [1] with 1037 participants found a 6% prevalence of childhood-onset ADHD and a 3% prevalence of adult-onset ADHD, with very little overlap. Moreover, other large-scale cohort studies [2, 3] have reported that a few patients met the diagnostic criteria for ADHD at both stages, suggesting that the causes of childhood-onset and adult-onset ADHD may differ and that the classification system for ADHD should be reconsidered. However, should the two forms of ADHD be regarded as separate disorders?

After careful consideration of these studies [1–3], we decided to focus on the intelligence quotient (IQ) and ADHD symptom scores of these patients. All studies [1–3] reported lower IQs in patients with childhood-onset ADHD. In the longitudinal twin study [2], the average IQ of patients who met the ADHD diagnostic criteria in both periods was significantly lower (mean, 88.0) than: that of patients whose ADHD diagnosis was not maintained in adulthood (mean, 93.0), that of patients diagnosed with adult-onset ADHD (mean, 96.9), and that of the control group (mean, 101.4). Therefore, low intelligence could be a major factor in symptom expression in childhood-onset ADHD. This suggests that low intelligence, and thus low latent social adaptation ability, may cause the early manifestation of ADHD symptoms, leading to a high likelihood of an ADHD diagnosis. Patients diagnosed with adult-onset ADHD had above average IQ scores and executive functioning during childhood, even though they tended to have ADHD symptoms [1, 2]. Thus, their social adaptation abilities may have masked ADHD behavioral characteristics, complicating the diagnosis [2].

Similar to the studies discussed above, neuroimaging studies have also elucidated the characteristics of each group. Only children with ADHD showed shrinkage in regions of the brain cortex [4], possibly because the disorder affects brain maturation [5]. Among patients with symptoms persisting into adulthood, fractional anisotropy was lower than that in an unaffected control group [6]. These results suggest that structural abnormalities are present in patients whose symptoms persist into adulthood [4, 5], but not in patients whose symptoms do not [6].

**Fig. 1:**
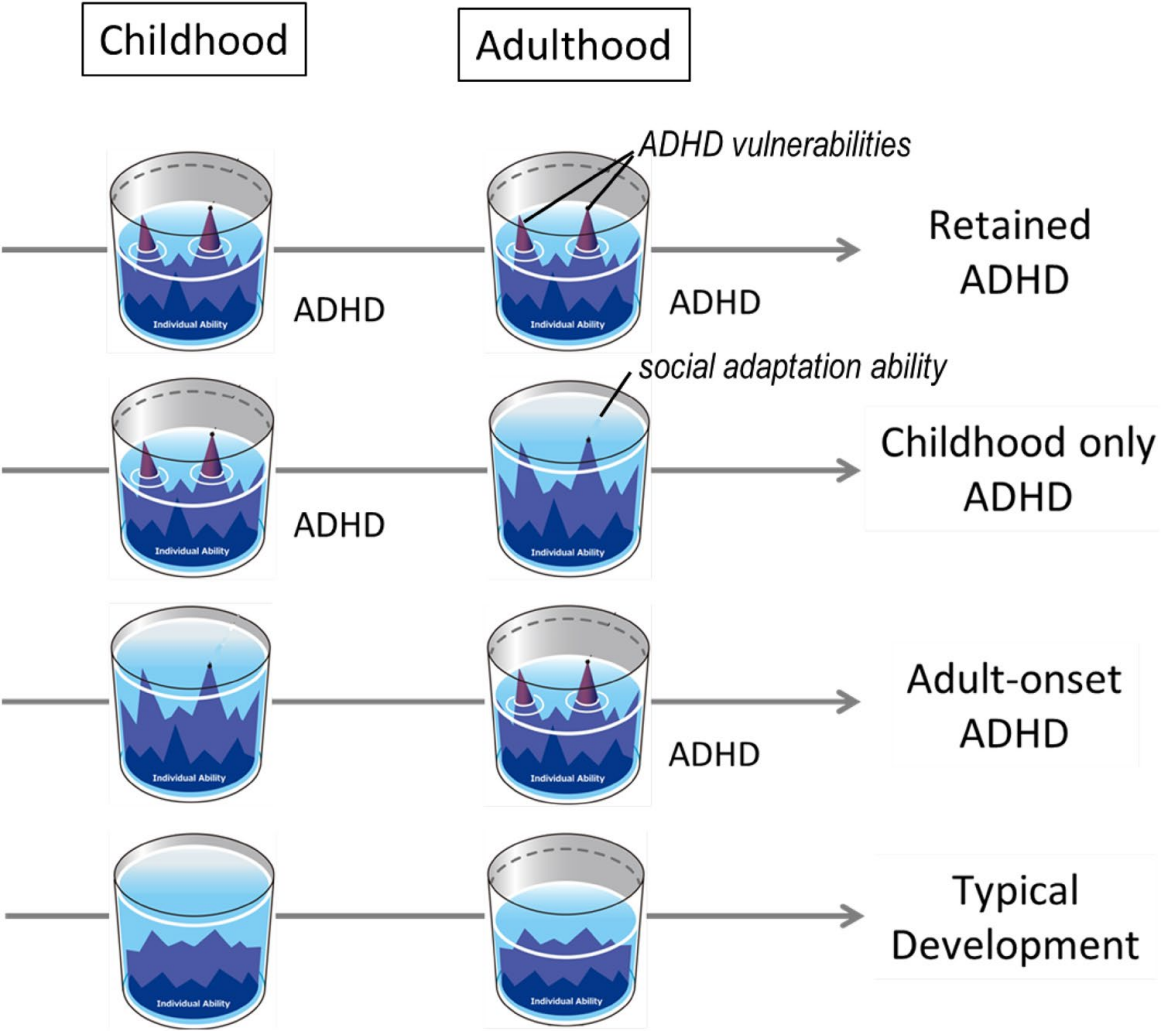
Social adaptation ability and manifestation of attention-deficit hyperactivity disorder (ADHD) symptoms. When social adaptation abilities are insufficient, adaptation difficulties cause the expression of latent ADHD characteristics, leading to a diagnosis. In cases where ADHD persists into adulthood, patients’ weaknesses are always exposed (Row 1). In cases of childhood-onset ADHD, if environmental support enables patients to achieve the required level of social adaptation, weaknesses can be hidden, improving adaptation in daily life, such that the disorder is no longer obvious (Row 2). If weaknesses that remained hidden in childhood due to adequate intelligence or environmental support are exposed in adulthood due to stress or other factors, adult-onset ADHD may be diagnosed (Row 3). If there are no major relative weaknesses in latent ability, weak areas and other factors will not be exposed even if these individuals experience stress (Row 4)

We considered the relationship between the expression of ADHD symptoms and social adaptation ability (Fig. 1). Individuals with neurodevelopmental disorders show differences in individual ability and areas of skill. For example, consider that the mind is represented as a glass and social adaptation ability as the contained water. If a patient’s latent intelligence is high, the volume of water is large; however, if the patient has structural brain impairments or low intelligence, the volume may be low. Moreover, imagine that environmental factors can influence water levels. In individuals with typical development, even if the water level declines during periods of high demand of social interaction with peers or severe stress, their vulnerabilities may not be as exposed as those of individuals with neurodevelopmental disorders. In contrast, in individuals with neurodevelopmental disorders, when this water level drops, their vulnerabilities are easily exposed. During these times, they have difficulty adapting in their areas of weakness, and the symptoms of their neurodevelopmental disorder become apparent.

Possibly, in cases of adult-onset ADHD, the patients had a good social environment during childhood, with strong family support, a stable school environment to which they could adapt, or sufficient intelligence to compensate for their weaknesses. This may be why they were not prone to adaptation difficulties at that stage [2]. However, during adulthood, family and environmental support diminishes, and they must act independently. Once their social adaptation abilities worsen, they are no longer able to compensate completely with their intellect. Therefore, their weaknesses are exposed, and they are consequently diagnosed with neurodevelopmental disorders. This implies that these patients are prone to developing neurodevelopmental disorders from birth. However, the opposite is also plausible. Even in cases where intelligence and brain structural effects cause childhood-onset ADHD with actualized symptoms, sometimes, the environment is adjusted to suit the unique characteristics of the patients and psychosocial support is provided. In these cases, the patients’ water levels could rise, their weaknesses could become less noticeable, self-respect may develop, neurodevelopmental disorder symptoms could weaken, and the diagnostic criteria could cease to describe them. However, other symptoms may be retained [2]. The characteristic areas of vulnerability in ADHD are not always apparent but could be temporal or conditional.

Hence, if individuals with and without neurodevelopmental disorders are both exposed to comparable stress, the former are more likely to experience difficulties in adapting. The difference between childhood-onset and adult-onset ADHD could be whether the difficulties in adaptation that are characteristic of neurodevelopmental disorders occur in childhood or adulthood. It, therefore, seems logical to regard these as forms of the same disease rather than as separate disorders [7, 8]. We are awaiting further data related to the genetic and biological backgrounds of ADHD patients [4–6].
